# Limited impact of neonatal or early infant schedules of 7-valent pneumococcal conjugate vaccination on nasopharyngeal carriage of *Streptococcus pneumoniae* in Papua New Guinean children: A randomized controlled trial

**DOI:** 10.1016/j.vacrep.2016.08.002

**Published:** 2016-12

**Authors:** Celestine Aho, Audrey Michael, Mition Yoannes, Andrew Greenhill, Peter Jacoby, John Reeder, William Pomat, Gerard Saleu, Pioto Namuigi, Suparat Phuanukoonnon, Heidi Smith-Vaughan, Amanda J. Leach, Peter Richmond, Deborah Lehmann, for the Neonatal Pneumococcal Conjugate Vaccine Trial Study Team

**Affiliations:** a Papua New Guinea Institute of Medical Research, PO Box 60, Goroka 441 EHP, Papua New Guinea; b Child Health Division, Menzies School of Health Research, PO Box 41096, Casuarina, Northern Territory 0811, Australia; c School of Applied and Biomedical Sciences, Federation University, Churchill, Victoria, Australia; d Telethon Kids Institute, The University of Western Australia, Perth, PO Box 855, West Perth, WA 6872, Australia; e Burnet Institute, 85 Commercial Road, Melbourne, Vic 3004, Australia; f The Walter and Eliza Hall Institute, 1G Royal Parade, Parkville, Vic 3052, Australia; g School of Paediatrics and Child Health, The University of Western Australia, Perth Western Australia, Australia

**Keywords:** Papua New Guinea, *Streptococcus pneumoniae*, Carriage, 7-Valent pneumococcal conjugate vaccine, Pneumococcal serotype, Neonate

## Abstract

*Streptococcus pneumoniae* is a leading cause of pneumonia, the most common cause of childhood death. Papua New Guinean children experience high rates of nasopharyngeal pneumococcal colonization within weeks of birth, predisposing them to pneumococcal disease. In a trial to determine the safety and immunogenicity of early infant vaccination with 7-valent pneumococcal conjugate vaccine (7vPCV), we investigated the impact of early schedules on pneumococcal carriage.

Infants were randomized at birth to receive 7vPCV in a 0–1–2-month (n = 101) or a 1–2–3-month (n = 105) schedule or no 7vPCV (n = 106). All children received 23-valent pneumococcal polysaccharide vaccine at age 9 months. We cultured nasopharyngeal swabs (NPS) collected at ages 1, 2, 3, 4 weeks and 3, 9, 18 months, and middle ear discharge if present. Pneumococcal serotypes were identified by the Quellung reaction.

A total of 1761 NPS were cultured. The prevalence of pneumococcal carriage was 22% at 1 week of age, rising to 80% by age 3 months and remained >70% thereafter, with high-density carriage in 42% of pneumococcus-positive samples. We identified 63 different serotypes; 43% of isolates from controls were 13vPCV serotypes. There were no significant differences in 7vPCV serotype carriage between 7vPCV recipients and controls at any age (22% vs. 31% at 9 months, p = 0.2). At age 9 months the prevalence of non-7vPCV carriage was 17% higher in 7vPCV recipients (48%) than in controls (25%, p = 0.02). More non-7vPCV serotypes were isolated from ear discharge in 16 7vPCV recipients than from 4 controls (48% vs. 25%, p = 0.13).

The limited impact of neonatal or accelerated infant 7vPCV schedules on vaccine serotype carriage is probably due to the early onset of dense carriage of a broad range of pneumococcal serotypes. While serotype-independent pneumococcal vaccines are needed in high-risk populations, the underlying environmental factors and sources of infection must be investigated.

http://clinicaltrials.gov/ct2/show/NCT00219401.

## Highlights

• High rates of dense pneumococcal carriage begin soon after birth in Papua New Guinea.
• Highly diverse range of pneumococcal serotypes is carried in the first month of life.
• Early 7vPCV schedules have limited impact on pneumococcal vaccine type carriage in PNG.

## 1 Introduction

Pneumonia is the most common cause of death in children worldwide [1]. *Streptococcus pneumoniae* (the pneumococcus) is a major cause of pneumonia as well as other invasive diseases such as meningitis and bacteraemia. It is also responsible for non-invasive diseases such as otitis media (OM) which can result in hearing loss [2]. Nasopharyngeal carriage of *S. pneumoniae* predisposes individuals to pneumococcal diseases; early carriage in infants is associated with increased risk of OM and is found in populations with a high incidence of invasive pneumococcal disease (IPD) [3], [4]. In the highlands of Papua New Guinea (PNG), colonization of the nasopharynx starts within weeks of birth, with all infants acquiring *S. pneumoniae* by 3 months of age and high carriage rates being maintained throughout childhood [4], [5], [6]. Pneumonia remains the most common cause of death and reason for hospitalization in childhood in PNG [7], [8]. Mortality due to acute lower respiratory infections (ALRI), in particular pneumonia, was reported in 1999 to be highest in the first year of life, with 56% of deaths in children under the age of five years occurring before six months of age [9].

Pneumococcal conjugate vaccines (PCVs) have been used to combat pneumococcal disease in infants. A protein carrier is linked to an otherwise T cell-independent polysaccharide antigen to evoke an immunological response and enhance long-term memory to serotypes included in the vaccines. The introduction and widespread use of PCVs in first world countries has resulted in a significant decline in nasopharyngeal carriage and IPDs caused by vaccine serotypes in children under the age of 2 years. PCVs also indirectly protect unvaccinated people through herd immunity [10], [11], [12]. Encouraging results have also been seen in third world settings where PCVs have been trialled [13], [14]. PCV given at ages 6, 10 and 14 weeks in third world countries and 2, 4 and 6 months with a booster in the second year of life in first world countries are immunogenic and reduce nasopharyngeal carriage of pneumococcal serotypes included in PCVs [10], [15]. In addition, a pneumococcal polysaccharide vaccine has previously been shown to reduce pneumonia mortality in PNG children when given between the ages of 6 months and 5 years [16]. However, in populations where carriage occurs within weeks of birth and where morbidity and mortality due to ALRI are highest in the first 6 months of life, interventions at a very young age are necessary. Hence, a trial evaluating an accelerated schedule including a neonatal PCV dose was carried out to assess the safety and immunogenicity of the seven-valent PCV (7vPCV) in children living in the Asaro Valley, Eastern Highlands Province, PNG. We have previously reported that neonatal and early infant immunization with 7vPCV is safe and immunogenic [17]. We now aim to describe the effect of 7vPCV on nasopharyngeal carriage of pneumococci and pneumococcal serotypes when given either in a 0–1–2 or 1–2–3-month schedule. We also look at the effect of 7vPCV on pneumococci isolated from ear discharge swabs of children with tympanic membrane perforation (TMP) due to OM.

## 2 Methods

A detailed description of the study site and population, process of recruitment, assent and consent, enrolment, randomisation method, allocation concealment, study staff and participant blinding, laboratory staff blinding, and participant characteristics by allocated group, immunization and follow-up for primary outcomes are reported elsewhere [17], [18]. A brief overview is given below.

### 2.1 Study design

The neonatal pneumococcal conjugate vaccine trial was carried out between 2005 and 2009. It was an open-label randomized controlled trial of 7vPCV (Wyeth/Pfizer; serotypes 4, 6B, 9V, 14, 18C, 19F & 23F) given at the ages of 0, 1 and 2 months (neonatal group) or at 1, 2 and 3 months (infant group) and a group that received no 7vPCV (control group). Study design and procedures are reported elsewhere [18]. All the children received other vaccinations according to the PNG Expanded Programme on Immunization [18], [19] and 23-valent pneumococcal polysaccharide vaccine (PPV, Merck & Co, Inc; serotypes 1, 2, 3, 4, 5, 6B, 7F, 8, 9N, 9V, 10A, 11A, 12F, 14, 15B, 17F, 18C, 19F, 19A, 20, 22F, 23F and 33F) was given at 9 months of age. Participants were followed up to the age of 18 months.

Participant recruitment is reported in Pomat et al. [17]. Inclusion criteria for enrolment were: the intention to remain in the study area for at least 2 years, a birth weight > 2000 grams, no acute neonatal infection, no severe congenital abnormality, and born in Goroka General Hospital (GGH) or brought to GGH within 24 h of birth. Children of mothers known to be HIV-positive were excluded.

### 2.2 Nasopharyngeal swab collection

Infants were seen either at home or at the PNG Institute of Medical Research (PNGIMR) clinic at ages 1, 2, 3 and 4 weeks and 3, 9 and 18 months. Nasopharyngeal swabs (NPS, rayon-tipped swabs with metal shaft. Medical Wire and Equipment, Wiltshire, England) were collected and processed according to WHO recommended procedures for pneumococcal carriage studies [20]. The swab was inserted into the nasopharynx until resistance was met and then rotated for five seconds. Immediately following collection, the swab was placed in 1 ml of skim milk tryptone glucose glycerol broth (STGGB) [20]. The swabs were kept cool in an insulated container with ice packs and taken to the PNGIMR bacteriology laboratory within 2 h and stored at −80 °C until processed.

Ear discharge swabs (Amies Copan, Interpath Services, Australia) were collected from children who had purulent ear discharge on examination. Ear discharge swabs were placed directly into 1 ml STGGB and transported and stored as for NPS.

### 2.3 Culture of nasopharyngeal swabs and ear swabs

Approximately 12% of NPS (235/1996) were lost following a freezer thaw (details in results section). The remaining NPS were cultured using standard bacteriological procedures [20] by laboratory staff blinded to PCV group. 10 μl aliquots of the NPS in STGGB were streaked onto horse blood agar, chocolate agar, gentamicin blood agar (5 μg/ml) and bacitracin chocolate agar (300 μg/ml). Plates were incubated overnight (18–24 h) at 37 °C in 5% CO_2_-enriched atmosphere. Bacterial growth was quantified on the plate as follows: 0, no growth; 1, <20 bacterial colonies; 2, 20–50 bacterial colonies; 3, 50–100 bacterial colonies; 4, confluent growth on the primary streak of the plate; 5, confluent growth on the primary streak of the plate and colonies on the second streak; 6, confluent growth in the secondary zone and colonies in the third zone. A quantification code of 1–3 was considered low density while 4–6 was considered high density. Four suspected morphologically distinct individual colonies of pneumococci on the primary plate (α-haemolytic low convex, plateau or draughtsman-shaped colonies) were picked and tested for optochin (5 μg ethylhydrocupreine, Oxoid, Australia) sensitivity. A zone of growth around the optochin disc of ⩾14 mm diameter was considered positive for *S. pneumoniae*. The bile solubility test was done on optochin-resistant colonies (<14 mm) to confirm *S. pneumoniae*.

### 2.4 Serotyping

Pneumococcal serotyping was done on all pneumococcal isolates by the Quellung reaction with antisera from Statens Serum Institut, Denmark. Serotyping on all 4 subcultured colonies was performed with all identified serotypes reported once per sample.

Relevant serogroups in the 7vPCV, namely 6, 9, 18, 19 and 23 were factor-typed (to the serotype level) along with the non-7vPCV serogroup 7. Not all factor sera were available at the time of study.

A representative sample (n = 134) of typed isolates was sent to Queensland Public Health laboratory for factor typing and serotype confirmation by the Quellung reaction: all serotyping results were concordant.

### 2.5 Statistical analysis

The primary endpoint for analysis was nasopharyngeal carriage rates of any *S. pneumoniae,* 7vPCV serotypes (VT: 4, 6B, 9V, 14, 18C, 19F, 23F) and non-7vPCV (NVT) serotypes. Vaccine-related serotypes were included with NVT. 23PPV serotypes and non-23PPV serotypes (all other) are reported where appropriate. Results were aggregated according to serotype to calculate the prevalence and proportions of individual serotypes among all serotypeable isolates. Non-serotypeable pneumococci are reported separately to the serotypeable isolates.

Chi-squared tests were used to assess between group differences in prevalence of VT and NVT serotypes at 1–4 weeks of age and at 3, 9 and 18 months. If prevalence rates or serotype distribution did not differ significantly between infant and neonatal groups, these were combined (vaccinated) for comparisons with controls.

### 2.6 Ethical considerations

Ethical clearance for the study was obtained from the PNG Medical Research Advisory Committee and from the Princess Margaret Hospital for Children Ethics Committee in Perth, Australia. The trial is registered at ClinicalTrials.gov under registration number NCT00219401 (http://clinicaltrials.gov/ct2/show/NCT00219401).

## 3 Results

### 3.1 Study population characteristics

Of the 448 mothers who assented for their children to be recruited into the study, 318 subsequently consented and were randomized to neonatal (n = 104), infant (n = 105) and control cohorts (n = 109). Six newborns were subsequently excluded on medical grounds. Details of loss to follow-up and protocol violations have been described elsewhere [18]. There were two deaths during the study period that were unrelated to study vaccines and 14 protocol violations relating to allocated immunization schedule [17]. No parent refused collection of NPS from their child at any time point. Supplementary Table 1 shows the distribution of collected samples that were cultured by age and group allocation. The proportion of samples that could not be cultured ranged from 7% to 18% with no significant differences between allocated groups or over time, although there were fewer thawed samples at ages 9 and 18 months than in younger children. Of the 312 children enrolled in the study, 309 (99 in neonatal, 104 in infant and 106 in control cohorts) had at least 1 sample collected during routine follow-up visits that was available for culture; 80% had culture data available from 5 or more samples with no significant difference between groups. Of the NPS cultured there was a lower proportion from male participants in the neonatal group (231/552, 42%) than in either the infant vaccination group (349/609, 57%) or controls (379/600, 63%) (Chi-squared = 55.73, p < 0.0001, 2df). Nevertheless, the gender distribution at each follow-up visit was consistent with the gender distribution of study participants reported previously [17], [18].

### 3.2 Age-specific prevalence of pneumococcal carriage by allocated group

**Fig. 1:**
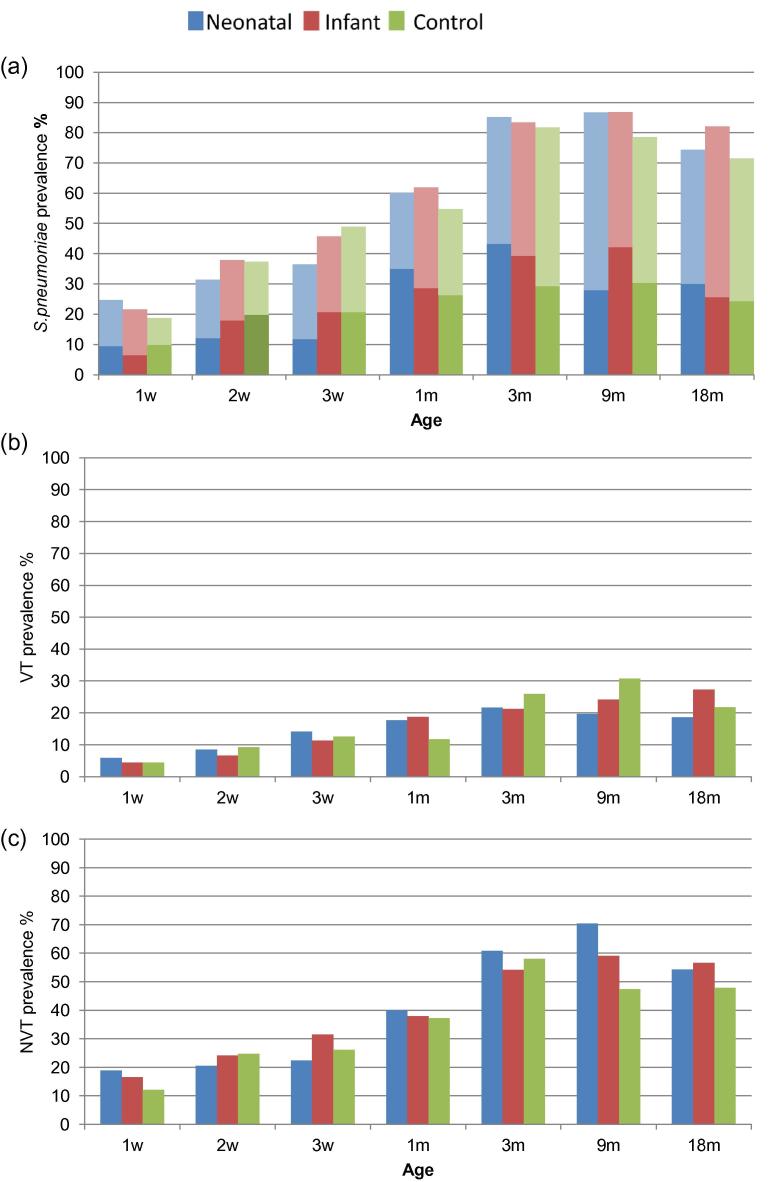
(a) Prevalence of *S. pneumoniae* carriage, (b) VT (7vPCV) carriage and (c) NVT (non-7vPCV) carriage by age according to vaccine cohort. Dark bars in (a) indicate prevalence of high density carriage.

We cultured a total of 552, 609 and 600 NPS collected during routine follow-up visits of children in the neonatal, infant and control groups, respectively (Table 1). At 1 week of age, 22% (n = 58) of children carried *S. pneumoniae*, increasing to 59% (n = 152) at 1 month of age and peaking at 9 months when 84% (n = 199) of children carried *S. pneumoniae* (Table 1). There were no significant differences in overall pneumococcal carriage rates between the neonatal, infant and control cohorts at any time point (Fig. 1a). Overall, 42% of pneumococcus-positive samples were high density, and there were no differences in density of carriage between neonatal, infant and control cohorts (Fig. 1a). The prevalence of non-serotypeable *S. pneumoniae* (failing to react with omni serum) was 8% of all samples.

**Table 1:** Prevalence of carriage of *Streptococcus pneumoniae* (Any Spn), high density *S. pneumoniae* (HD Spn), 7vPCV serotypes (VT Spn) and non-7vPCV serotypes (NVT Spn) in neonatal, infant and control groups by age.

Vaccine group	Age of routine visits								
		1 week	2 weeks	3 weeks	4 weeks	3 months	9 months	18 months	Total
		n/nps (%)⁎	n/nps (%)	n/nps (%)	n/nps (%)	n/nps (%)	n/nps (%)	n/nps (%)	n/nps (%)
Neonatal PCV	Any Spn	21/85 (24.7)	26/83 (31.3)	31/85 (36.5)	48/80 (60.0)	63/74 (85.1)	65/75 (86.7)	52/70 (74.3)	306/552 (55.4)
	HD Spn	8/85 (9.4%)	10/83 (12.0)	10/85 (11.8)	28/80 (35.0)	32/74 (43.2)	21/74 (28.4)	21/70 (30.0)	130/551 (23.6)
	VT Spn	5/85 (5.9)	7/83 (8.4)	12/85 (14.1)	14/79 (17.7)	16/74 (21.6)	14/71 (19.7)	13/70 (18.6)	81/547 (14.8)
	NVT Spn	16/85 (18.8)	17/83 (20.5)	19/85 (22.4)	32/80 (40.0)	45/74 (60.8)	50/71 (70.4)	38/70 (54.3)	217/548 (39.6)

Infant PCV	Any Spn	20/93 (21.5)	36/95 (37.9)	42/92 (45.7)	52/84 (61.9)	70/84 (83.3)	72/83 (86.7)	64/78 (82.1)	356/609 (58.5)
	HD Spn	6/93 (6.5)	17/95 (17.9)	19/92 (20.7)	24/84 (28.6)	33/84 (39.3)	35/83 (42.2)	20/78 (25.6)	154/609 (25.3)
	VT Spn	4/91 (4.4)	6/91 (6.6)	10/88(11.4)	15/80 (18.8)	17/80 (21.3)	20/83 (24.1)	21/77 (27.3)	93/590 (15.8)
	NVT Spn	15/91 (16.5)	22/91 (24.2)	28/89 (31.5)	30/79 (38.0)	45/83 (54.2)	49/83 (59.0)	43/76 (56.6)	232/592 (39.2)

Control	Any Spn	17/91 (18.7)	34/91 (37.4)	45/92 (48.9)	52/95 (54.7)	67/82 (81.7)	62/79 (78.5)	50/70 (71.4)	327/600 (54.5)
	HD Spn	9/91 (9.9)	18/91 (19.8)	19/92 (20.7)	25/95 (26.3)	24/82 (29.3)	24/79 (30.4)	17/70 (24.3)	136/600 (22.7)
	VT Spn	4/91 (4.4)	8/87 (9.2)	11/88 (12.5)	11/94 (11.7)	21/81 (25.9)	24/78 (30.8)	15/69 (21.7)	94/588 (16.0)
	NVT Spn	11/91 (12.1)	22/89 (24.7)	23/88 (26.1)	35/94 (37.2)	47/81 (58.0)	37/78 (47.4)	33/69 (47.8)	208/590 (35.3)

Different Denominators in VT Spn and NVT Spn are due to the exclusion of indeterminate serotypes like 6NF (NF = no factor type) and 6A/C (not discriminated between 6A and 6C).

⁎ n/nps (%) = number positive/number of nasopharyngeal swabs cultured (%).

All available *S pneumoniae* isolates (Supplementary Table 2) were serotyped to the serogroup level, and for VT, most were grouped to the serotype level. Fifty-five serogroup 6 (5.5%) isolates, one serogroup 18 (0.1%), twelve isolates from serogroup 19 (1.2%) and six isolates of serogroup 23 (0.6%) were not resolved to serotype level and were not included in subsequent analyses or counted towards the total serotypes. We were unable to discriminate 6C from 6A for 48 isolates due to non-availability of factor type 6C in the laboratory in PNG. However, for 41 isolates typed as 6A in PNG, 6C was differentiated from 6A by PCR at the Menzies School of Health Research in Darwin. Fig. 1b shows the prevalence of VT and Fig. 1c, the prevalence NVT carriage in the neonatal, infant and control cohorts at routine follow-up visits. At 3 months of age, VT carriage prevalence was 22, 21 and 26 percent and NVT carriage was 61, 54 and 58 percent in the neonatal, infant and control cohorts respectively (Table 1). There was no significant difference in VT or NVT carriage prevalence between vaccinated and unvaccinated cohorts before the age of 9 months or at age 18 months (For example 3 months: VT Chi-squared 0.38, p = 0.54, NVT Chi-squared = 0.001 p = 0.97; 18 months VT Chi-squared = 0.003, p = 0.96; NVT Chi-squared = 0.82, p = 0.37.). However, differences were noted at age 9 months: the prevalence of NVT carriage was significantly higher in the neonatal and infant groups combined than in controls (64% (99/154) vs. 47% (37/78) (Chi-squared = 5.39, 1df, p = 0.02, Table 1, Fig. 1c). Prevalence of VT carriage at 9 months was lowest in the neonatal group (20% (14/71)), 24% (20/83) in the infant group, and 31% (24/78) among controls (Chi-squared 7vPCV recipients to controls = 1.64, 1df, p = 0.2, Table 1, Fig. 1b).

### 3.3 Serotype-specific data

Sixty-three distinct *S. pneumoniae* serogroups/serotypes were identified in this study; 53 different serotypes/serogroups were identified within the first month of life. The overall distribution of serotypes in the neonatal, infant and control groups, all ages combined, is provided in Supplementary Table 2. The top serotypes in each group were: Neonatal 19F (11%), 15 (10%), 19A (8%), 23F (5%), 6A/C (4%), 6B (3%), 16 (3%), 21 (3%) and 35 (3%); Infant 19F (11%), 15 (6%), 6B (4%), 16 (4%), 6A/C (4%), 19A (4%), 35 (4%), 34 (3%) and 6A (3%); Control 23F (8%), 19F (8%), 15 (7%), 6A/C (7%), 19A (7%), 14 (5%), 35 (3%), 3 (3%), 6B (3%) and 16 (3%). At age 9 months, when there was the greatest impact of PCV on VT/NVT carriage, there were no significant differences between 7vPCV recipients and controls for individual serotypes, although serotype 19F tended to be isolated more often among controls than 7vPCV recipients (Supplementary Table 3, Chi-squared 7vPCV recipients to controls = 2.14, 1df, p = 0.14).

**Fig. 2:**
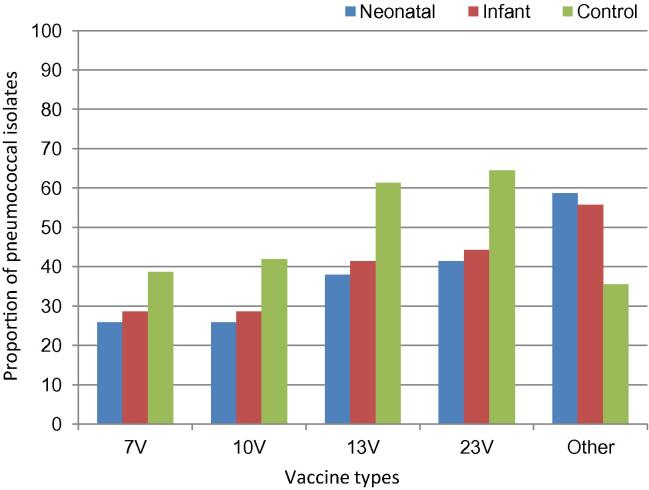
Proportion of pneumococcal serotypes in nasopharyngeal swabs covered by 7vPCV, 10vPCV, 13vPCV and 23PPV, and those not present in any licensed vaccine (‘Other’), according to vaccine cohort at 9 months of age.

Overall, the proportion of fully characterised serotypeable pneumococcal carriage isolates covered by the 7vPCV in the neonatal, infant and control cohorts was 25%, 26% and 28% respectively, while the more recently licenced 13vPCV (which includes additional serotypes 1, 3, 5, 7F, 6A, 19A) covered 38%, 36% and 43% of pneumococcal isolates, respectively. In the control cohort at age 9 months 39% of serotypeable isolates were covered by the 7vPCV while 61% were covered by 13vPCV (Fig. 2). At 9 months, 7vPCV recipients had significantly higher carriage of serotypes not included in any licensed pneumococcal vaccine (depicted as “Other” in Fig. 2) than controls; neonatal 59%; infant 56%; control 36% (Chi squared = 6.9, 1df, p = 0.01).

Simultaneous carriage of multiple serotypes in pneumococcus-positive samples (for which serotypes were fully differentiated) was detected in 7% (20/293), 9% (28/325) and 11% (32/289) in the neonatal, infant and control cohorts respectively.

### 3.4 Pneumococcal isolation rates from middle ear discharge

Swabs of middle ear discharge were collected from one or both ears during 49 episodes of acute otitis media with perforation or chronic suppurative otitis media (13 in neonatal group, 20 in infant group, and 16 in controls) occurring in 36 children (11 neonatal, 14 infant, 11 control). The children were all aged >3.5 months and those in the neonatal and infant groups had completed immunization according to the allocation group. Pneumococcal isolation rates from ear discharge were 46% (6/13), 65% (13/20) and 50% (8/16) in neonatal, infant and control groups, respectively, with no significant difference between groups. Three of the 27 pneumococcal ear discharge isolates (all in the infant group) could not be revived for serotyping. Three 7vPCV serotypes (3/16; 19%) were isolated from ear discharge collected from controls and one each in neonatal (1/13; 8%) and infant (1/20; 5%) cohorts (neonatal + infant vs. control; Fisher’s Exact test, p = 0.31). Non-7vPCV serotypes were more commonly isolated from ear discharge collected from children who had received 7vPCV than from controls, but this was not significant (46%, 6/13 neonatal; 50%, 10/20 infant; 25%, 4/16 controls, (neonatal + infant (48%) vs. control (25%); Fisher’s Exact test 0.10, p = 0.13). Notably, serotype 19A was isolated from 21% (7/33) of ear swabs collected from 7vPCV recipients compared with 6% (1/16) among controls (neonatal + infant vs. control; Fisher’s Exact test p = 0.25). NVTs accounted for 86% of serotyped pneumococci in the neonatal group, 91% in the infant group and 57% in controls (neonatal + infant vs. control; Fisher’s Exact test p = 0.11). Of the 26 pneumococcal isolates with available serotype data, the serotypes identified in the neonatal group were 3 (n = 2), 5, 18B, 19A (n = 2, one of which was isolated from same swab as aforementioned serotype 3) and 23F. Serotypes in the infant group were 11C (n = 2), 19A (n = 5), 19F, 35 (n = 2) and 45. In controls the serotypes were 5, 6B, 18C, 19A, 19F, 20 (together with a non-serotypeable isolate) and 24.

## 4 Discussion

Our study is one of only two neonatal PCV studies worldwide. The Kenyan neonatal study [21] reported carriage at two time points in two PCV vaccinated groups whereas we report carriage at 7 time points and include an unvaccinated control group, with all infants in the study receiving an additional booster dose of 23PPV at 9 months.

We found early and high *S. pneumoniae* nasopharyngeal carriage increasing with age across the three cohorts of infants irrespective of vaccine schedule. The lack of a significant difference in overall prevalence of pneumococci between vaccinated and control groups was also observed in Kenyan infants and other high risk populations in Africa and Fiji where prevalence of carriage is high [21], [22], [23]. Furthermore, pneumococcal carriage was dense in a high proportion of pneumococcus-positive samples in our study regardless of their PCV vaccination status and high density is associated with increased risk of disease [24]. Further studies investigating the effect of PCV on density of carriage using quantitative PCR for total bacterial load and species-specific load are needed in PNG.

The underlying mechanisms that foster and sustain the elevated carriage in this population are unclear. However, possible sources of respiratory pathogens could be contact with densely colonised older siblings [25], the child’s mother [6] and adults with chronic lung disease which is a prevalent condition in the highlands of PNG [26]. Overcrowding is an important risk factor and was found to be the strongest predictor of carriage in Australian Aboriginal children [25]. Overcrowding in the highlands of PNG is common and is also likely to be a factor driving high nasopharyngeal carriage transmission in this population. It is common for a family, often including members of the extended family, to live together in a traditional highland house, which typically comprises just one communal room with smoke from a wood fire in the centre of the room [27]. Exposure to environmental tobacco smoke and wood smoke from burning biomass fuel, a popular form of fuel for cooking due to its affordability, may also contribute to high carriage rates by enhancing nasopharyngeal colonization [28], [29]. In an indoor air pollution study, Kirarock recorded significantly higher levels of coarse particulate matter (PM_10_) released from burning of biofuel in traditional highland houses than in semi-permanent and permanent houses [27]. Hand hygiene can also play a role in the acquisition and transmission of different serotypes in children. Aboriginal children living in remote communities were 3 times more likely to have nasal discharge and 23 times more likely to have hand contamination with both *S. pneumoniae* and *H. influenzae* than children attending urban day care centres [30]. More needs to be done to increase public health awareness on healthy living lifestyle and improving living conditions especially in the rural areas.

A total of sixty-three different pneumococcal serotypes/serogroups were detected over the duration of the study illustrating the high diversity of serotypes present in this population. This is likely to be an underestimate as factor sera were not available for all serogroups. Pneumococcal isolates with indeterminate serotype, like 6A/C, were not counted as separate serotypes. Factor typing was not available in our previous studies in PNG; therefore this is the first study to report any serotype-specific carriage data in Papua New Guinean children. Overall, the high diversity of serotypes in this population may limit the impact of limited valency conjugate vaccines.

In this study there was a reduction in VT carriage in the vaccinated cohorts at 9 months of age, although not statistically significant, and no difference in carriage of any pneumococcus between vaccinated and unvaccinated cohorts. However, the prevalence of NVT carriage at 9 months was significantly higher in 7vPCV recipients than in controls suggesting some impact of PCV on the pneumococcal carriage populations in vaccinated children. It is likely that the limited effect of 7vPCV on VT carriage is due to the high density of carriage and diversity of serotypes circulating, with relatively low prevalence of 7vPCV types; in the control cohort only 39% of carried isolates at 9 months of age were 7vPCV types.

We have previously reported immunogenicity to PCV7 in this trial [17]. Geometric mean concentrations (GMTs) of serotype-specific IgG levels for all VT were above the cut-off value of 0.35 μg/mL for protection from IPD. At age 4 months antibody GMTs were remarkably similar to those reported in Kenya following the standard 6–10–14-week PCV schedule commonly used in third world settings and also following the neonatal schedule trialled in Kenya [17], [21]. The more conservative value of 1 μg/mL was more discriminatory as less than half of the samples at age 9 months had a serotype-specific antibody titre ⩾1 μg/mL for serotypes 4, 9V, 18C and 23F. However, it is noteworthy that antibody titers were high at age 9 months for those serotypes that were more commonly isolated at age 3 months (6B, 14 and 19F), suggesting that carriage boosts the antibody response. The proportion of samples with antibody titers ⩾1 μg/mL in our study was higher for all VTs than among recipients of the standard EPI schedule in Kenya [21]. To our knowledge there is no established correlate for protection against pneumococcal carriage although 5 μg/mL was found by Goldblatt et al. to be protective against serotype 14 carriage in adults in the United Kingdom [31]. While theoretically administration of PPV at age 9 months may affect memory B cells numbers at age 18 months, we have found circulating VT-specific memory B-cells are present at 18 months and are not different to unvaccinated controls at age 3–5 years [32]. The relationship between carriage and immune response to pneumococci requires further investigation.

Our study had several limitations. Firstly, the study was originally powered to detect group differences in immune response [17], [18] and, consequently, some of the potentially important observed differences in secondary outcomes such as carriage prevalence, do not reach statistical significance. For example, the key finding of a difference in prevalence of VT at 9 months of age between vaccinated (22%) and unvaccinated (31%) groups would require a sample size of 278 per group to have 80% power to be detected as statistically significant (p < 0.05). This would involve the recruitment of approximately 350 infants per group whereas only around 100 per group were actually enrolled. Secondly, the time points selected for sampling may not have been optimal to detect impact of PCV on carriage: age 3 months may have been too early to see an effect on VT carriage while 9 months may have been too late. We saw a non-significant difference in VT carriage between PCV recipients and controls at age 9 months, but the impact of PCV on VT carriage might have already peaked at age six months.

Not all samples could be cultured as samples were lost following a freezer failure. Nevertheless 87% of samples (n = 1761) were cultured and for 80% of children we had culture data from 5 or more samples per child. Furthermore, we did not have factor type sera for all serogroups and therefore could not serotype all isolates (although some additional factor typing was available on isolates characterised in Australia). Hence the serotype diversity is an underestimate. Finally, in view of the wide diversity of serotypes and the consequent small number of isolates across the serotypes, we could not look at the impact of 7vPCV on individual serotypes.

The high and varied diversity in serotypes limits PCV coverage and in turn increases the likelihood of replacement serotypes in carriage and disease. Following the rollout of the 13vPCV in PNG in 2014, it is vital to closely monitor carriage and invasive pneumococcal disease serotypes to observe direct and population herd effects of vaccine on serotype distribution. Serotype-independent vaccines are needed to protect against pneumococcal disease irrespective of capsular serotype, but there is also a need to address the underlying environmental factors and to determine the sources of infection in children.

## Financial disclosure

This project was funded by an International Collaborative Project Grant of the Wellcome Trust, United Kingdom (071613/Z/03/Z) and the Australian National Health and Medical Research Council (NHMRC #303123).

DL received funding from an NHMRC Program grant (#353514) and a Project Grant (#572590). The funders had no role in study design, data collection and analysis, decision to publish, or preparation of the manuscript. AJL is funded by an NHMRC Research Fellowship (#1020561). CA is currently funded through an Australia Awards Scholarship administered by the Australian Department of Foreign Affairs and Trade.

## Authors’ contributions

The study was conceived and designed by DL, PCR, JR, with microbiological input from AJL. DL, PCR, JR obtained the funding to conduct the study. AG oversaw the microbiology. CA, AM, MY conducted microbiological analysis and reviewed all microbiology data. JR oversaw the implementation of the clinical trial and provided administrative support. AJL and HSV provided microbiological expertise in preparation of the manuscript. SP supervised enrolment, clinical follow-up, specimen collection and field work. PN and GS did clinical follow-up and collected all NPS. PJ and DL analysed the data. CA and DL drafted the manuscript. All authors have critically read and approved the final version of the manuscript.

## Conflicts of Interest

AG, DL and WP have received research support through a Pfizer Investigator Initiated Grant. DL has been a member of the GlaxoSmithKline Australia Pneumococcal-*Haemophilus influenzae*-Protein D conjugate vaccine Advisory Panel, has received support from Pfizer Australia and GSK Australia to attend conferences, and has received an honorarium from Merck Vaccines to give a seminar at their offices in Pennsylvania and to attend a conference. WP received a travel grant from Pfizer Australia to attend the 8th International Symposium on Pneumococci and Pneumococcal Diseases (ISPPD) in 2012. AJL has received research support through Investigator Initiated Grants from Pfizer and GlaxoSmithKline. PR is a member of the Vaccine Trials Group, which has received funding from vaccine manufacturers, including Pfizer, Sanofi Pasteur, BioCSL, and GlaxoSmithKline. Other authors declare no conflict of interest.
