# Supplementary material for: Limited impact of neonatal or early infant schedules of 7-valent pneumococcal conjugate vaccination on nasopharyngeal carriage of Streptococcus pneumoniae in Papua New Guinean children: A randomized controlled trial
Source: Vaccine Rep. 2016 Dec;6:36–43. doi: 10.1016/j.vacrep.2016.08.002 (PMC5446595; doi:10.1016/j.vacrep.2016.08.002)
Supplement: Supplementary data 1 [file mmc1.doc]

| Age | Vaccine groups | | | | | |  | |
| --- | --- | --- | --- | --- | --- | --- | --- | --- |
|  | Neonatal | | Infant | | Control | | Total | |
|  | cultured/ collected | percent | cultured/ collected | percent | cultured/collected | percent | cultured/collected | percent |
| 1 wk | 85/100 | (85%) | 93/105 | (89%) | 91/106 | (86%) | 269/311 | (86%) |
| 2 wks | 83/96 | (86%) | 95/104 | (91%) | 91/106 | (86%) | 269/306 | (88%) |
| 3 wks | 85/96 | (89%) | 92/103 | (89%) | 92/104 | (88%) | 269/303 | (89%) |
| 4 wks | 80/94 | (85%) | 84/103 | (82%) | 95/103 | (92%) | 259/300 | (86%) |
| 3 mths | 74/89 | (83%) | 84/97 | (87%) | 82/91 | (90%) | 240/277 | (87%) |
| 9 mths | 75/81 | (93%) | 83/92 | (90%) | 79/86 | (92%) | 237/259 | (92%) |
| 18 mths | 70/78 | (90%) | 78/87 | (90%) | 70/75 | (93%) | 218/240 | (91%) |
| Total | 552/634 | (87%) | 609/691 | (88%) | 600/671 | (89%) | 1761/1996 | (88%) |

**Supplementary Table 1** Nasopharyngeal swabs (NPS) cultured (which had not thawed) of the number of NPS collected (culture/collected (percent)) at different ages in neonatal, infant and control groups.
