# Supplementary material for: Limited impact of neonatal or early infant schedules of 7-valent pneumococcal conjugate vaccination on nasopharyngeal carriage of Streptococcus pneumoniae in Papua New Guinean children: A randomized controlled trial
Source: Vaccine Rep. 2016 Dec;6:36–43. doi: 10.1016/j.vacrep.2016.08.002 (PMC5446595; doi:10.1016/j.vacrep.2016.08.002)
Supplement: Supplementary data 3 [file mmc3.docx]

**Supplementary Table 3** Pneumococcal serotypes/serogroups and proportion of serotypeable pneumococci isolated from the nasopharynx in neonatal, infant and control cohorts at age 9 months.

| Serotype | Neonatal | | Infant | | Control | | Total | |
| --- | --- | --- | --- | --- | --- | --- | --- | --- |
|  | N | % | N | % | N | % | N | % |
| 2 | 1 | 1.4 | 0 | 0 | 0 | 0 | 1 | 0.5 |
| 3 | 1 | 1.4 | 1 | 1.4 | 2 | 3 | 4 | 1.9 |
| 4 | 0 | 0 | 0 | 0 | 1 | 1.5 | 1 | 0.5 |
| 6A | 3 | 4.3 | 3 | 4.2 | 4 | 6.1 | 10 | 4.8 |
| 6A/C | 7 | 10 | 2 | 2.8 | 3 | 4.5 | 12 | 5.8 |
| 6B | 2 | 2.9 | 4 | 5.6 | 3 | 4.5 | 9 | 4.3 |
| 6C | 1 | 1.4 | 0 | 0 | 1 | 1.5 | 2 | 1 |
| 6NF | 2 | 2.9 | 0 | 0 | 0 | 0 | 2 | 1 |
| 7F | 0 | 0 | 0 | 0 | 2 | 3 | 2 | 1 |
| 8 | 0 | 0 | 2 | 2.8 | 0 | 0 | 2 | 1 |
| 9L | 2 | 2.9 | 1 | 1.4 | 0 | 0 | 3 | 1.4 |
| 10 | 2 | 2.9 | 3 | 4.2 | 0 | 0 | 5 | 2.4 |
| 10A | 0 | 0 | 0 | 0 | 1 | 1.5 | 1 | 0.5 |
| 10F | 0 | 0 | 2 | 2.8 | 0 | 0 | 2 | 1 |
| 11 | 2 | 2.9 | 2 | 2.8 | 0 | 0 | 4 | 1.9 |
| 11A | 1 | 1.4 | 0 | 0 | 0 | 0 | 1 | 0.5 |
| 11C | 1 | 1.4 | 0 | 0 | 0 | 0 | 1 | 0.5 |
| 12 | 2 | 2.9 | 0 | 0 | 0 | 0 | 2 | 1 |
| 13 | 0 | 0 | 0 | 0 | 2 | 3 | 2 | 1 |
| 14 | 3 | 4.3 | 4 | 5.6 | 3 | 4.5 | 10 | 4.8 |
| 15 | 8 | 11.4 | 5 | 6.9 | 5 | 7.6 | 18 | 8.7 |
| 16 | 3 | 4.3 | 6 | 8.3 | 0 | 0 | 9 | 4.3 |
| 17 | 1 | 1.4 | 0 | 0 | 0 | 0 | 1 | 0.5 |
| 18A | 1 | 1.4 | 1 | 1.4 | 1 | 1.5 | 3 | 1.4 |
| 18C | 1 | 1.4 | 0 | 0 | 0 | 0 | 1 | 0.5 |
| 18F | 0 | 0 | 0 | 0 | 1 | 1.5 | 1 | 0.5 |
| 19A | 3 | 4.3 | 5 | 6.9 | 6 | 9.1 | 14 | 6.7 |
| 19C | 1 | 1.4 | 0 | 0 | 1 | 1.5 | 2 | 1 |
| 19F | 7 | 10 | 7 | 9.7 | 12 | 18.2 | 26 | 12.5 |
| 19NF | 2 | 2.9 | 0 | 0 | 0 | 0 | 2 | 1 |
| 21 | 3 | 4.3 | 0 | 0 | 0 | 0 | 3 | 1.4 |
| 22 | 0 | 0 | 1 | 1.4 | 0 | 0 | 1 | 0.5 |
| 22A | 0 | 0 | 1 | 1.4 | 1 | 1.5 | 2 | 1 |
| 23A | 1 | 1.4 | 0 | 0 | 1 | 1.5 | 2 | 1 |
| 23B | 1 | 1.4 | 0 | 0 | 1 | 1.5 | 2 | 1 |
| 23F | 2 | 2.9 | 5 | 6.9 | 5 | 7.6 | 12 | 5.8 |
| 23NF | 1 | 1.4 | 0 | 0 | 1 | 1.5 | 2 | 1 |
| 24 | 0 | 0 | 1 | 1.4 | 0 | 0 | 1 | 0.5 |
| 25 | 0 | 0 | 1 | 1.4 | 0 | 0 | 1 | 0.5 |
| 28 | 1 | 1.4 | 0 | 0 | 0 | 0 | 1 | 0.5 |
| 29 | 0 | 0 | 2 | 2.8 | 1 | 1.5 | 3 | 1.4 |
| 31 | 1 | 1.4 | 1 | 1.4 | 0 | 0 | 2 | 1 |
| 32 | 0 | 0 | 1 | 1.4 | 0 | 0 | 1 | 0.5 |
| 33 | 0 | 0 | 1 | 1.4 | 0 | 0 | 1 | 0.5 |
| 33B | 0 | 0 | 3 | 4.2 | 1 | 1.5 | 4 | 1.9 |
| 33F | 0 | 0 | 0 | 0 | 1 | 1.5 | 1 | 0.5 |
| 34 | 0 | 0 | 2 | 2.8 | 2 | 3 | 4 | 1.9 |
| 35 | 0 | 0 | 4 | 5.6 | 3 | 4.5 | 7 | 3.4 |
| 35B | 1 | 1.4 | 0 | 0 | 0 | 0 | 1 | 0.5 |
| 36 | 0 | 0 | 0 | 0 | 1 | 1.5 | 1 | 0.5 |
| 37 | 0 | 0 | 1 | 1.4 | 0 | 0 | 1 | 0.5 |
| 40 | 1 | 1.4 | 0 | 0 | 0 | 0 | 1 | 0.5 |
| 48 | 1 | 1.4 | 0 | 0 | 0 | 0 | 1 | 0.5 |
| Total | 10 | 100 | 72 | 100 | 66 | 100 | 208 | 100 |

6A/C refers to serotype 6A isolates that were not further discriminated for serotype 6C

NF Not Factor typed

Characterization of pneumococci was limited to serogroup where factor typing antisera were not available.
